# Supplementary material for: Cellular organization in lab-evolved and extant multicellular species obeys a maximum entropy law
Source: eLife. 2022 Feb 21;11:e72707. doi: 10.7554/eLife.72707 (PMC8860445; doi:10.7554/eLife.72707)
Supplement: Transparent reporting form [file elife-72707-transrepform1.docx]

***eLife’s* transparent reporting form**

We encourage authors to provide detailed information *within their submission* to facilitate the interpretation and replication of experiments. Authors can upload supporting documentation to indicate the use of appropriate reporting guidelines for health-related research (see [EQUATOR Network](http://www.equator-network.org/%20)), life science research (see the [BioSharing Information Resource](https://biosharing.org/)), or the [ARRIVE guidelines](http://www.plosbiology.org/article/info:doi/10.1371/journal.pbio.1000412) for reporting work involving animal research. Where applicable, authors should refer to any relevant reporting standards documents in this form.

If you have any questions, please consult our Journal Policies and/or contact us: [editorial@elifesciences.org](mailto:editorial@elifesciences.org).

**Sample-size estimation**

- You should state whether an appropriate sample size was computed when the study was being designed
- You should state the statistical method of sample size computation and any required assumptions
- If no explicit power analysis was used, you should describe how you decided what sample (replicate) size (number) to use

Please outline where this information can be found within the submission (e.g., sections or figure legends), or explain why this information doesn’t apply to your submission:

Information about sample sizes can be found in the Methods and Materials sections. In these sections, there is detailed information about how many samples (organisms) were imaged and measured and how many simulations were computed. Detailed information about experimental measurements can be found in the subsections “Scanning electron microscopy” for snowflake yeast organisms, and “Imaging Volvox” for the volvocine algae. Information about the number of each simulation can be found in the “Simulation methods” subsection. Information about choosing sample sizes can also be found in the “Scanning electron microscopy” subsection.

**Replicates**

- You should report how often each experiment was performed
- You should include a definition of biological versus technical replication
- The data obtained should be provided and sufficient information should be provided to indicate the number of independent biological and/or technical replicates
- If you encountered any outliers, you should describe how these were handled
- Criteria for exclusion/inclusion of data should be clearly stated
- High-throughput sequence data should be uploaded before submission, with a private link for reviewers provided (these are available from both GEO and ArrayExpress)

Please outline where this information can be found within the submission (e.g., sections or figure legends), or explain why this information doesn’t apply to your submission:

Information regarding replicates can be found in the Methods section of the paper. In that section, there is detailed information about how many organisms were measured for both snowflake yeast and volvocine algae, and there is information regarding the number of independent simulations of various growth morphologies. We include information about any outliers in the Methods section, too. All independent measurements in this paper can be regarded as technical replications rather than biological replications, since generally the organisms studied are grown together in the same sample tube, under the same conditions, and imaged together. Therefore, in this paper we do not distinguish between biological and technical replication events.

**Statistical reporting**

- Statistical analysis methods should be described and justified
- Raw data should be presented in figures whenever informative to do so (typically when N per group is less than 10)
- For each experiment, you should identify the statistical tests used, exact values of N, definitions of center, methods of multiple test correction, and dispersion and precision measures (e.g., mean, median, SD, SEM, confidence intervals; and, for the major substantive results, a measure of effect size (e.g., Pearson's r, Cohen's d)
- Report exact p-values wherever possible alongside the summary statistics and 95% confidence intervals. These should be reported for all key questions and not only when the p-value is less than 0.05.

Please outline where this information can be found within the submission (e.g., sections or figure legends), or explain why this information doesn’t apply to your submission:

There is detailed information about statistics (such as root mean square deviation from predictions, mean and standard deviations, and more) in every figure caption and main text description of the figures in this paper. We describe the number of measurements, measurement quality, and any outliers in the Methods sections.

(For large datasets, or papers with a very large number of statistical tests, you may upload a single table file with tests, Ns, etc., with reference to sections in the manuscript.)

**Group allocation**

- Indicate how samples were allocated into experimental groups (in the case of clinical studies, please specify allocation to treatment method); if randomization was used, please also state if restricted randomization was applied
- Indicate if masking was used during group allocation, data collection and/or data analysis

Please outline where this information can be found within the submission (e.g., sections or figure legends), or explain why this information doesn’t apply to your submission:

We did not group data into experimental groups in this paper. Similarly, there was no masking used during data collection nor data analysis.

**Additional data files (“source data”)**

- We encourage you to upload relevant additional data files, such as numerical data that are represented as a graph in a figure, or as a summary table
- Where provided, these should be in the most useful format, and they can be uploaded as “Source data” files linked to a main figure or table
- Include model definition files including the full list of parameters used
- Include code used for data analysis (e.g., R, MatLab)
- Avoid stating that data files are “available upon request”

Please indicate the figures or tables for which source data files have been provided:

We provide source data for the following:

Cartesian coordinates of cell centers for experimental measurements of snowflake yeast.

Cartesian coordinates of cell centers for experimental measurements of Volvox carteri.

Cartesian coordinates of cell centers for simulated snowflake yeast, and measured polygon areas of somatic cells for simulated volvox organisms.

Cartesian coordinates of cell centers for aggregative simulations, both with and without size polydispersity.

Cartesian coordinates of cell centers for simulations of surface-bound cells with planned apoptosis.

Cartesian coordinates of cell centers for simulations of prescribed tree-like growth with varying noise strengths.

Furthermore, we provide code for running any of the simulations described in the paper.

Together, this provides the raw data and/or the raw MatLab simulation code to generate data from every main figure in the paper.
